# Comparative pharmacoeconomic analysis of rituximab and traditional tacrolimus regimens in membranous nephropathy in China

**DOI:** 10.3389/fphar.2023.1309930

**Published:** 2024-01-08

**Authors:** Li Zeng, Huihui Chen, Heng Xiang, Mengru Zeng, Mi Zhou, Chongqing Tan, Hong Liu, Guochun Chen

**Affiliations:** ^1^ Department of Nephrology, The Second Xiangya Hospital of Central South University, Changsha, China; ^2^ Hunan Key Laboratory of Kidney Disease and Blood Purification, The Second Xiangya Hospital of Central South University, Changsha, China; ^3^ Clinical Immunology Research Center of Central South University, Changsha, China; ^4^ Department of Ophthalmology, The Second Xiangya Hospital of Central South University, Changsha, China; ^5^ Department of Pharmacy, The Second Xiangya Hospital of Central South University, Changsha, China

**Keywords:** Markov model, membranous nephropathy, pharmacoeconomic analysis, rituximab, tacrolimus

## Abstract

**Background:** Rituximab (RTX) is a monoclonal antibody that selectively targets CD20 and is frequently used in the treatment of membranous nephropathy (MN). Analysis of the therapeutic efficacy and safety of RTX in treating MN in practice and a comparative pharmacoeconomic analysis of the RTX and traditional tacrolimus (TAC) regimens can provide valuable insights to aid decision-making by the government and relevant medical insurance departments.

**Methods:** We conducted a statistical analysis of medical records from patients diagnosed with MN who underwent RTX treatment between 1 January 2019 and 1 January 2023. The TAC data were obtained from the clinical literature. The efficacy rates and incidence of adverse effects (AEs) were calculated to compare the efficacy and safety of RTX and TAC. Based on the patient’s disease status, we developed a Markov model to compare the total cost, remission rate, and incremental cost-effectiveness ratio (ICER) of the two regimens. Both univariate and probability sensitivity analyses were performed to validate the stability of the developed model.

**Results:** The RTX group enrolled 53 patients with MN, and the 12-month overall efficacy rate was not significantly different from that of the TAC group with 35 patients (86.79% vs. 71.4%, *p* = 0.0131); however, the relapse rate was significantly lower in the RTX group (3.77% vs. 22.8%, *p* = 0.016). The RTX group demonstrated no severe AEs (SAEs), while the TAC group demonstrated six cases of SAEs, including 4 cases of severe pneumonia, 1 case of lung abscess and 1 case of interstitial lung disease, accounting for 7.89% of traditional tacrolimus-treated patients. The baseline analysis results revealed that over a 5-year post-treatment period, RTX increased quality-adjusted life years (QALYs) by 0.058 and costs by ¥7,341. Assuming three times the 2022 domestic gross domestic product as the willingness-to-pay (WTP) threshold per QALY, the ICER of RTX compared to TAC was ¥124,631.14/QALY, which is less than the WTP threshold of ¥257,094/QALY, indicating that RTX treatment is approximately two times more cost-effective compared to TAC.

**Conclusion:** The current analysis indicates that despite the expensive unit price of RTX, it remains a cost-effective treatment option for MN compared to TAC.

## 1 Introduction

Chronic Kidney Disease (CKD) represents a substantial global public health concern, with China experiencing a particularly high prevalence rate of 10.8% within its adult populace (Zhang et al., 2012). Within the range of glomerulopathy etiologies, primary membranous nephropathy (PMN) is notably recognized as one of the leading causes of CKD, especially contributing to adult nephrotic syndrome (NS) (Xu et al., 2016; Ronco et al., 2021). PMN demonstrates a diverse disease trajectory, with 40%–50% of patients experiencing spontaneous remission, while 30%–50% progress to NS, eventually developing end-stage renal disease (ESRD) within a 5–10-year span (Schieppati et al., 1993; Troyanov et al., 2004; Hofstra et al., 2013). This variability in disease progression leads to a considerable healthcare burden on governmental resources. Given its straightforwardness and practicality, the assessment of disease activity and prognosis in PMN typically relies on measuring proteinuria levels and renal function. A decrease in proteinuria often signifies a reduced risk of renal decline (Dahan et al., 2017; Fervenza et al., 2019; Fernández-Juárez et al., 2021).

Following the identification of the M-type phospholipase A2 receptor as a pathogenic target antigen, PMN is now classified as an autoimmune disease (Beck et al., 2009). Consequently, treatment for progressive PMN primarily focuses on immunosuppression, often involving a combination of glucocorticoids (GCs) and immunosuppressive agents such as cyclophosphamide (CTX) and tacrolimus (TAC) (Ruggenenti et al., 2017; Rojas-Rivera et al., 2022). Although the modified Ponticelli regimen, which combines GCs and CTX, has demonstrated treatment success, it is associated with considerable side effects such as infection risk increase, osteoporosis, diabetes mellitus, infertility, and malignancy (Ponticelli et al., 1995; van den Brand et al., 2017). TAC, a calcineurin inhibitor (CNI), effectively suppresses T cell activation and proliferation. While the efficacy of TAC compared to CTX therapy in PMN remains a subject of ongoing debate, certain reports indicate that TAC may lead to comparable outcomes (Zhu et al., 2017; Zou et al., 2019). Nevertheless, nearly half of the patients relapse post-TAC regimen withdrawal, thus posing challenges in determining the ideal cessation timing (Ramachandran et al., 2017; Rojas-Rivera et al., 2023). Additionally, the nephrotoxicity associated with TAC therapy impacts patient adherence to the treatment (Praga et al., 2007; Knops et al., 2023).

Rituximab (RTX) is a monoclonal antibody that targets the CD20 antigen on B cells, inducing B-cell apoptosis and triggering complement-mediated cytotoxicity and antibody-dependent cytotoxicity, thereby exerting immunosuppressive effects (Deng and Xu, 2023). Given its pharmacological properties, RTX is extensively used in cancer treatment and has recently been employed for autoimmune diseases (Boye et al., 2003; Buch et al., 2011; Gan et al., 2021). Recent clinical investigations have indicated the efficacy of RTX for MN treatment, demonstrating its comparable or even superior therapeutic effects over other immunotherapies and mitigating concerns related to the safety of traditional immunosuppressive therapies (Dahan et al., 2019; Fervenza et al., 2019; Seitz-Polski et al., 2019; Chan et al., 2020; Fernández-Juárez et al., 2021; Scolari et al., 2021; Chan et al., 2022).

Despite the promising efficacy and safety profile of RTX for PMN treatment, its high cost restricts widespread application in clinical settings, particularly in developing nations. Nonetheless, a pharmacoeconomic analysis should not confine itself merely to the cost of a drug; instead, it should take into account potential savings in other medical costs brought about by the drug’s therapeutic effects. A recent UK study suggested potential pharmacoeconomic advantages of RTX in MN treatment (Hamilton et al., 2018). With significant disparities in medical insurance systems between China and other countries, the necessity for domestic clinical data to investigate RTX’s pharmacoeconomic characteristics in treating MN is evident. In response, we undertook a comprehensive examination of the efficacy, safety, and cost-effectiveness of RTX and TAC in MN treatment, viewed from the perspective of China national health system. This study’s findings will offer valuable insights to healthcare providers and policymakers in China and other countries with similar healthcare structures, aiding the informed use of RTX and TAC in MN treatment.

## 2 Methods

### 2.1 Study approval

The study was reviewed and approved by the Human Research Ethics Committee of the Second Xiangya Hospital of Central South University (2019SNK1220000) and was carried out in accordance with the principles outlined in the World Medical Association Declaration of Helsinki. Written informed consent was obtained from all participants at the time of admission.

### 2.2 Study design

An extensive literature survey affirmed that there exist no studies providing a direct pharmacoeconomic comparison between rituximab and tacrolimus specifically within the Chinese demographic. A cost-effectiveness analysis was performed from the perspective of the Chinese health system by developing and using a Markov model. The primary outcomes were the efficacy, safety, and cost-effectiveness of RTX *versus* those of TAC at 5 years post-treatment. Our data were derived from two distinct sources: a real-world study and corresponding representative literature. Employing the cost-effectiveness framework, the principal measure of benefit was quantified by the quality-adjusted life year (QALY), with the results of the analysis being articulated as incremental cost-effectiveness ratios (ICERs) per QALY.

### 2.3 Study populations and interventions

#### 2.3.1 RTX cohort

**FIGURE 1:**
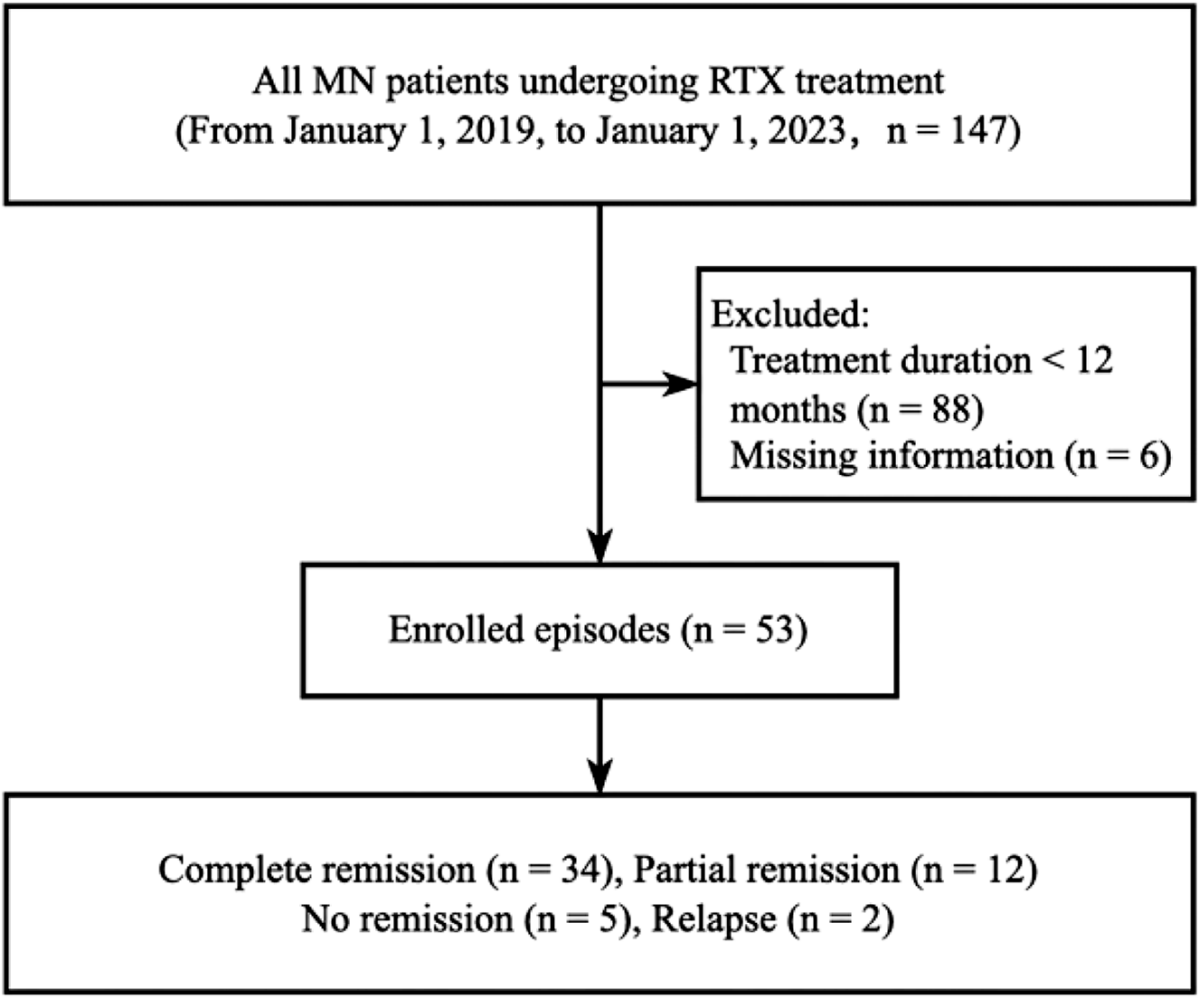
Derivation of the cohort.

The study population for RTX comprised all MN patients who received RTX treatment, as per the Hospital Information System (HIS) records of the Second Xiangya Hospital of Central South University, within the period from 1 January 2019, to 1 January 2023. Following the application of stringent filtering criteria, a total of 53 participants were retrospectively assessed. Inclusion criteria were set as follows: 1) biopsy-proven PMN diagnosis, accompanied by substantial proteinuria (>3.5 g/24 h), 2) patients who received RTX therapy (Hanlikang, 100 mg/10 mL/bottle, Shanghai Fuhong Hanlin Biopharmaceutical Co., Ltd.). Exclusion criteria were set as follows: 1) treatment duration less than 12 months (*n* = 88) and 2) incomplete information (*n* = 6). The data filtering process is depicted in Figure 1. The HIS system was utilized to extract demographic characteristics of the participants (such as age, gender, and onset time), diagnosis information, hospitalization status, RTX medication details, drug cost, post-treatment proteinuria to creatinine ratio (PCR), qualitative and quantitative proteinuria, and all Adverse Events (AEs) (Supplementary Table S1).

For the RTX group, the initial treatment protocol entailed administering Rituxan through intravenous injection at a weekly dose of 375 mg per square meter of body surface area. Patient evaluations were conducted at 1, 3, 6, 9, and 12 months following the commencement of treatment. In instances where proteinuria registered a reduction of at least 25% from the baseline at the 6-month mark without achieving complete remission (CR), a second round of RTX treatment was deemed necessary, irrespective of the CD19^+^ B cell count. The second course of RTX treatment administered at our center follows a full induction protocol. However, if CR was achieved post the 6-month treatment period, a second round of treatment was deemed unnecessary. On the other hand, if proteinuria registered a decrease of less than 25% after 6 months, the treatment was considered ineffective, and RTX was discontinued. Following the directives provided by the KDIGO, 2021 Clinical Practice Guideline (Radhakrishnan and Cattran, 2012) and our clinical practice experiences, the therapeutic effectiveness for MN is stratified into four categories depending upon clinical outcomes, including CR, partial remission (PR), no remission (NR), and relapse. The parameters defining therapeutic effectiveness are as shown in Supplementary Table S2.

#### 2.3.2 TAC cohort

Upon meticulous review and baseline information alignment of pertinent literature regarding TAC treatment for MN, the TAC data derived from the study entitled “Efficacy and safety of long-course tacrolimus treatment for idiopathic membranous nephropathy” (Di et al., 2018) was chosen as the control against the RTX-treated MN cohort. Baseline characteristics such as age and sex, as well as the number of patients with significant proteinuria in the TAC group, showed no substantial difference from the RTX group within the HIS. Despite a marked discrepancy in the onset time, it did not influence the analysis outcomes in this investigation, indicating a prolonged and more severe disease condition in the RTX group. The baseline features of both groups are illustrated in Table 1.

**TABLE 1:** Baseline characteristics of the RTX and TAC groups.

Characteristic	RTX group (*n* = 53)	TAC group (*n* = 35)	*p-*value
Age (year)	31.9 ± 16.8	47.9 ± 17.1	0.443
Female (n,%)	12 (22.64%)	13 (37.14%)	0.140
Onset time (month)	41.2 ± 61.5	11.6 ± 5.3	<0.001
Massive proteinuria (n,%)	42 (79.25%)	30 (85.71%)	0.441

Age, patients undergoing RTX or TAC treatment; Onset time, the period between the diagnosis of PMN and the initiation of treatment; RTX, rituximab; TAC, tacrolimus.

TAC treatment regimen was as described in the referenced study (Di et al., 2018). Initially, patients with MN were administered an oral combination therapy of TAC and prednisolone acetate tablets. The starting dosage of TAC was determined to be 0.1 mg/kg/day, administered every 12 h. Over the course of the 12-month follow-up, plasma concentrations of TAC were rigorously monitored—weekly during the first month and subsequently on a monthly basis—to ensure levels remained within the therapeutic window of 5–10 μg/L. After 6 months, the concentration of TAC was judiciously reduced to a maintenance range of 2–4 μg/L until the study’s conclusion at 12 months. Regarding prednisolone acetate, the initial dosage was set at 0.5 mg/kg/day, taken once daily. This dosage was sustained for the first 8 weeks, followed by a tapering of 5 mg/day every 4 weeks, culminating in a maintenance dose of 10 mg/day which was then continued through to the end of the study period.

### 2.4 Mathematical model

**FIGURE 2:**
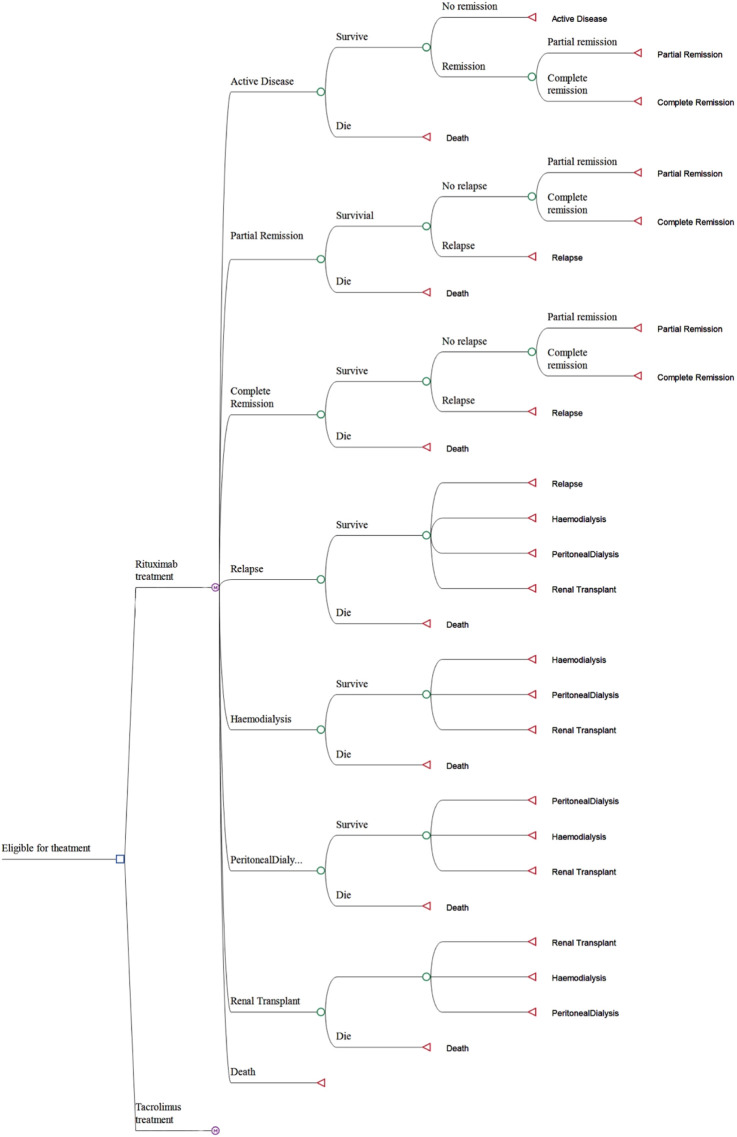
Model structure.

A Markov model was developed using the TreeAge Pro software (Tree-Age, Williamstown, Massachusetts, United States) to compute the costs and efficacy of both RTX and TAC treatment regimens. This model is composed of eight distinct states, which include active disease, complete remission, partial remission, relapse, hemodialysis, peritoneal dialysis, kidney transplant, and death. All patients in the study entered the model in the active disease state. They began treatment with either RTX or TAC in combination with steroid therapy. Following the onset of treatment, patients transitioned through various health states, and the model simulated a 5-year period subsequent to the patients receiving their initial dose of medication. The Markov model was divided into 30-day cycles, which is a duration brief enough to mimic the frequency of clinical events and treatment interventions without leading to the bias commonly associated with longer cycles (Chhatwal et al., 2016). The efficacy was evaluated through Quality-Adjusted Life Years (QALYs), and the cost and efficacy values were discounted at an annual rate of 5%. The Incremental Cost-Effectiveness Ratio (ICER) was derived from the Markov model and was employed to compare cost-effectiveness (Drummond et al., 2023). The primary cost-effectiveness outcomes of this study include the total cost, QALYs, and ICER. The Markov model is graphically presented in Figure 2.

### 2.5 Input parameters

#### 2.5.1 Transition probabilities, utility scores, and costing

The transition probabilities pertinent to distinct treatment phases, including active disease, CR, PR, relapse, hemodialysis, peritoneal dialysis, kidney transplant, and death, were derived from multiple research sources. These probabilities were subsequently converted into monthly values. The Markov model was then populated with these monthly transition probabilities. QALYs, which encapsulate the health status of a particular condition, assign a value of 1 to a year of perfect health and 0 to death. For the purposes of this study, utility scores were established at 0.73 QALYs for active disease and 0.86 QALYs for complete remission. These scores were derived from four separate studies, each exploring the quality of life at various stages of MN treatment. For patients with complete or partial remission, we used age- and sex-matched EQ-5D UK population norms, while the remaining utility values were derived by converting SF-36 values into EQ-5D utility scores. The model incorporates the cost of drugs (administration cost per cycle for the RTX group and control group that received TAC combined with prednisone), the cost of AEs associated treatment, as well as the expenses incurred after relapse, including post-relapse treatments such as peritoneal dialysis, hemodialysis, and kidney transplantation. The prices used in the model were derived from the HIS system of the hospital or published literature. Details of Markov model input parameter, along with their respective sources, are shown in Table 2.

**TABLE 2:** Details of Markov model input parameter.

Variable	Mean value	Range	Reference category	Distribution/Notes
Annual transition rate of states				
Peritoneal dialysis to hemodialysis	0.1633		Li et al. (2023)	
Hemodialysis to peritoneal dialysis	0.0032		Li et al. (2023)	
Incidence of dialysis	0.0002		Li et al. (2017)	
Incidence of hemodialysis	0.0002		Wong et al. (2020), Li et al. (2023)	
Incidence of peritoneal dialysis	0.00003		Wong et al. (2020), Li et al. (2023)	
Incidence of kidney transplantation	0.0794		Wong et al. (2020)	
Hemodialysis to death	0.0422		Li et al. (2023)	
Peritoneal dialysis to death	0.0388		Li et al. (2023)	
10-year mortality after kidney transplantation	0.425	0.35–0.50	Zou et al. (2023)	
Utility value				
Complete remission	0.8600	0.630–1.000	Kind et al. (1999)	Beta/age and sex matched
Partial remission	0.8600	0.630–1.000	Kind et al. (1999)	Beta/age and sex matched
Active disease	0.7380	0.422–1.000	Liborio et al. (2023)	Beta/SF-36 converted to EQ-5D
End-stage kidney disease	0.8000	0.650–0.940	Wyld et al. (2023)	Beta/SF-36 converted to EQ-5D
Conservative treatment	0.6200	0.360–0.890	Wyld et al. (2023)	Beta/SF-36 converted to EQ-5D
Hemodialysis	0.6800	0.530–0.820	Wyld et al. (2023)	Beta/SF-36 converted to EQ-5D
Peritoneal dialysis	0.7100	0.590–0.820	Wyld et al. (2023)	Beta/SF-36 converted to EQ-5D
Kidney transplantation	0.8200	0.740–0.900	Wyld et al. (2023)	Beta/SF-36 converted to EQ-5D
Death	0.0000	0.000	Hamilton et al. (2018)	
Incidence of AEs of the RTX group				
Rash	0.0377	0.03–0.045	Clinical data	Beta
Fever	0.0189	0.015–0.023	Clinical data	Beta
Gastrointestinal infection	0.0189	0.015–0.023	Clinical data	Beta
Pneumonia	0.0189	0.015–0.023	Clinical data	Beta
Incidence of AEs of the RTX group				
Severe pneumonia	0.1143	0.091–0.137	Di et al. (2018)	Beta
Lung abscess	0.0286	0.023–0.034	Di et al. (2018)	Beta
Interstitial pneumonia	0.0286	0.023–0.034	Di et al. (2018)	Beta
Hyperglycemia	0.1429	0.114–0.171	Di et al. (2018)	Beta
Cost of AEs (¥/time)				
Rash	44.3751	35.500–53.250	Clinical data	Gamma
Fever	33.3000	26.640–39.960	Clinical data	Gamma
Gastrointestinal infection	425.0545	340.043–510.064	Clinical data	Gamma
Pneumonia	7,400.0000	5920.000–8880.000	Clinical data	Gamma
Lung abscess	10,000.0000	8000.000–12000.000	Clinical data	Gamma
Interstitial pneumonia	4,000.0000	3200.000–4800.000	Clinical data	Gamma
Hyperglycemia	15.0000	12.000–18.000	Clinical data	Gamma
Direct medical costs				
Hemodialysis cost **(**¥/year**)**	40,678.0000	32542.400–48813.600	Li et al. (2023)	Gamma
Peritoneal dialysis cost **(**¥/year**)**	31,145.0000	24916.000–37374.000	Li et al. (2023)	Gamma
Kidney transplantation	10,278.8000	8223.040–12334.560	Hospital Authority (2017a)	Gamma
RTX [100 mg/10 mL*1] (vial)	1,366.2000	1093.000–1639.000	Clinical data	Gamma
Cost of TAC (6 months)				
Complete remission (¥)	9,976.0000	7980.800–11971.200	Wang et al. (2021)	Gamma
Partial remission (¥)	9,976.0000	7980.800–11971.200	Wang et al. (2021)	Gamma
No remission (¥)	10,626.0000	8500.800–12751.200	Wang et al. (2021)	Gamma
Relapse (¥)	10,626.0000	8500.800–12751.200	Wang et al. (2021)	Gamma
Methylprednisolone [4 mg*30] (table)	0.878	0.702–1.054	Clinical data	Gamma
Urinalysis (¥/test)	47.0000	37.60–56.40	Clinical data	Gamma
Rapid proteinuria test (¥/test)	44.0000	35.20–52.80	Clinical data	Gamma
Quantitative proteinuria test (¥/test)	9.0000	7.20–10.80	Clinical data	Gamma
Routine liver function test (¥/test)	55.0000	44.00–66.00	Clinical data	Gamma
Willing to pay threshold (¥/QALY)	257,094.0000		National Bureau of Statistics of China (2023)	
Discount rate	0.0500		Liu et al. (2011)	

#### 2.5.2 Willingness-to-pay threshold (WTP)

In accordance with the World Health Organization’s recommendation, the WTP threshold for this study was set using a benchmark of three times China’s gross domestic product *per capita*. This result in a WTP value of 257,094 (3 × 85,698) yuan per quality-adjusted life year (QALY), providing a standard for determining cost-effective strategies.

### 2.6 Sensitivity analyses

Key determinants of cost-effectiveness were identified via one-way deterministic sensitivity analyses for each variable incorporated in the analysis. Acceptable ranges for all variables were derived from their respective confidence intervals. Additionally, a probabilistic sensitivity analysis was undertaken, employing a Monte Carlo simulation with 100,000 iterations, to evaluate the influence of uncertainty across all transition probabilities, costs, and health utilities. The model was utilized to compute the likelihood that RTX would be deemed cost-effective over 1-, 2-, 3-, 4-, and 5-year time horizons.

## 3 Results

### 3.1 Effectiveness analysis

As presented in Table 3, therapeutic efficiency indicators were employed to determine the number of patients in the RTX group who achieved CR, PR, NR, and relapse. These quantities were then utilized to calculate respective rates. Differences between groups were evaluated using a Chi-square test. After a 12-month treatment period, out of the 53 patients in the RTX group, 34 achieved CR, 12 reached PR, 5 experienced NR, and 2 relapsed after CR. Comparatively, RTX appeared slightly more efficacious than TAC in treating membranous nephropathy after 12 months of treatment; however, the difference in overall remission between the two groups was not statistically significant (86.79% vs. 71.4%, *p* = 0.131). Nonetheless, the rate of CR was significantly higher in the RTX group than in the TAC group (64.15% vs. 22.86%, *p* < 0.001), and the relapse rate was lower (3.77% vs. 22.8%, *p* = 0.016). These results suggest that the RTX regimen may exhibit superiority over the TAC regimen in treating MN.

**TABLE 3:** Efficacy comparison of RTX and TAC for MN treatment.

Efficacy indicators	RTX group	TAC group	*p-*value
Episodes	53	35	
Complete remission (n,%)	34 (64.15%)	8 (22.86%)	<0.001
Partial remission (n,%)	12 (22.64%)	17 (48.57%)	0.011
No remission (n,%)	5 (9.43%)	10 (28.57%)	0.019
Relapse (n,%)	2 (3.77%)	8 (22.86%)	0.016
Overall (n,%)	46 (86.79%)	25 (71.43%)	0.131

Overall = Complete remission + Partial remission, MN: membranous nephropathy, RTX: rituximab, TAC: tacrolimus.

### 3.2 Safety analysis

As presented in Table 4, out of the 53 patients who received RTX therapy for MN, five individuals were subject to AEs, resulting in an AE occurrence rate of 9.43%. Specifically, two patients (3.77%) experienced a dermatological manifestation in the form of skin rash, one patient (1.89%) presented with fever, one patient (1.89%) developed gastrointestinal infection, and one patient (1.89%) was diagnosed with lung infection. In contrast, according to the referenced literature, of the 76 patients who were administered the TAC regimen, 11 individuals (14.47%) encountered AEs. In particular, six patients (7.89%) underwent SAEs, inclusive of four (5.26%) cases of grave pneumonia, which tragically led to the death of three (3.95%) patients. Additionally, there was one instance each (1.32%) of lung abscess and interstitial pneumonia. Moreover, five patients (6.58%) reported mild AEs, all of which correlated with elevated blood glucose levels.

**TABLE 4:** Adverse effect comparison of RTX and TAC for MN treatment.

Adverse effect	Episodes	Incidence (%)
RTX		
Rash	2	3.77
Fever	1	1.89
Gastrointestinal Infection	1	1.89
Pneumonia	1	1.89
Overall	5	9.43
TAC		
Hyperglycemia	5	5.26
Severe pneumonia	4	5.26
Lung abscess	1	1.32
Interstitial pneumonia	1	1.32
Overall	11	13.16

MN, membranous nephropathy; RTX, rituximab; TAC, tacrolimus.

### 3.3 Cost-effectiveness analysis

#### 3.3.1 ICER

Analyzing from the perspective of cumulative costs over a 5-year treatment period, the cost associated with RTX therapy was found to be higher than that of TAC regimen (¥178,006.0 vs. ¥170,665.2). However, the cumulative QALYs for RTX surpassed that of TAC (3.706 vs. 3.648). Thus, a straightforward comparison of the absolute cost-effectiveness of the two treatment regimens is not feasible. In light of this, the ICER was calculated for RTX in comparison to TAC, resulting in a value of ¥124,631 per QALY. Given the WTP threshold set at ¥257,094 per QALY, the RTX treatment therefore presents a more cost-effective option (Table 5).

**TABLE 5:** Cost-effectiveness comparison of RTX and TAC for MN treatment.

Year(s)	Cost	QALYs	Cumulative cost	Cumulative QALYs
RTX				
1	32,976.36	0.825	32,976.36	0.825
2	33,630.16	0.790	66,606.52	1.615
3	35,509.82	0.741	102,116.34	2.356
4	37,240.27	0.696	139,356.61	3.052
5	38,649.39	0.655	178,006.00	3.706
TAC				
1	30,676.87	0.817	30,676.87	0.817
2	31,047.08	0.778	61,723.94	1.595
3	34,461.23	0.726	96,185.17	2.321
4	36,675.36	0.682	132,860.54	3.003
5	37,804.71	0.644	170,665.25	3.648

MN, membranous nephropathy; QALYs, Quality-adjusted life-years; RTX, rituximab; TAC, tacrolimus.

#### 3.3.2 Sensitivity analyses

**FIGURE 3:**
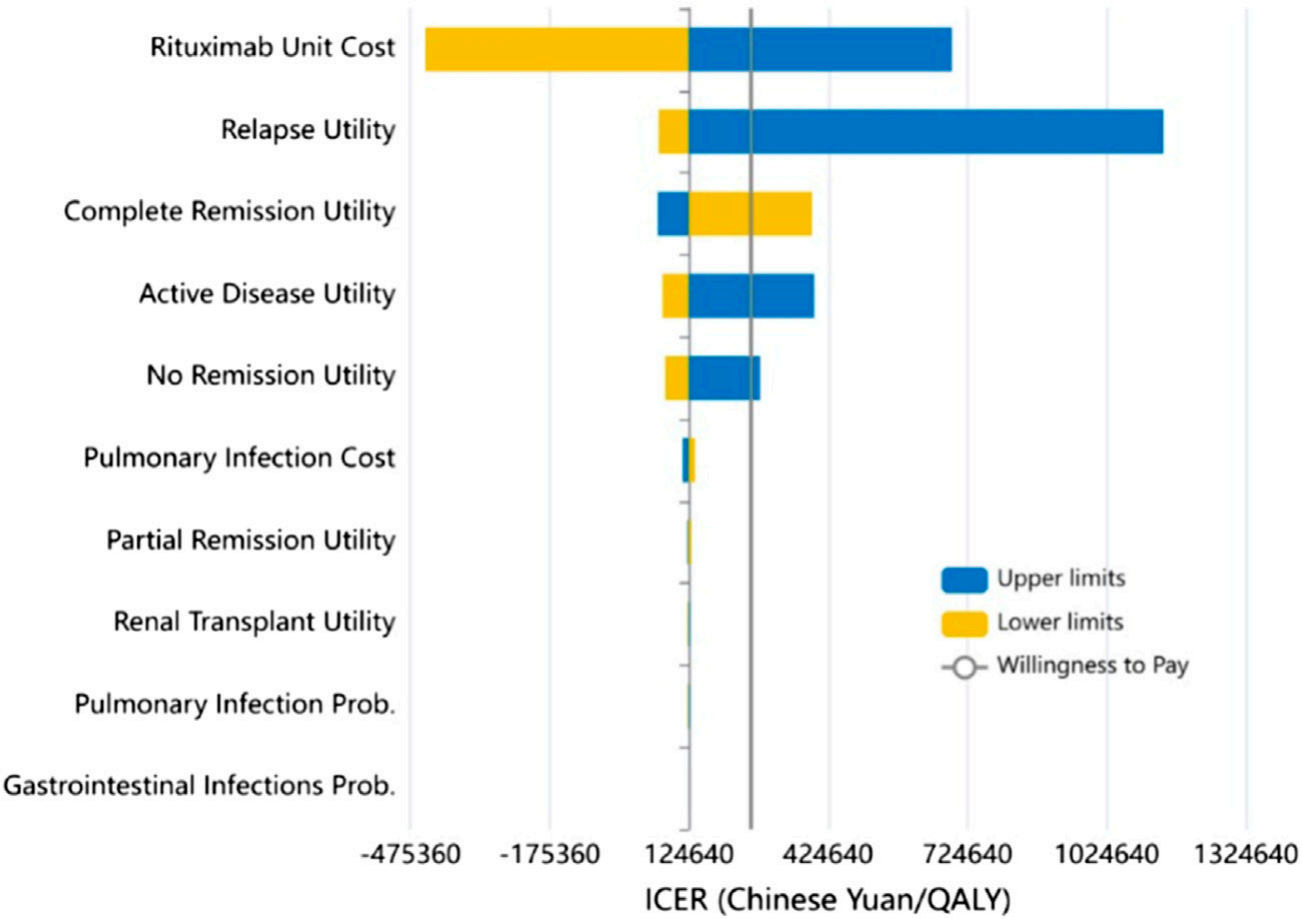
Results of the one-way sensitivity analysis (Tornado diagram) and incremental cost-effectiveness ratio (ICER).

**FIGURE 4:**
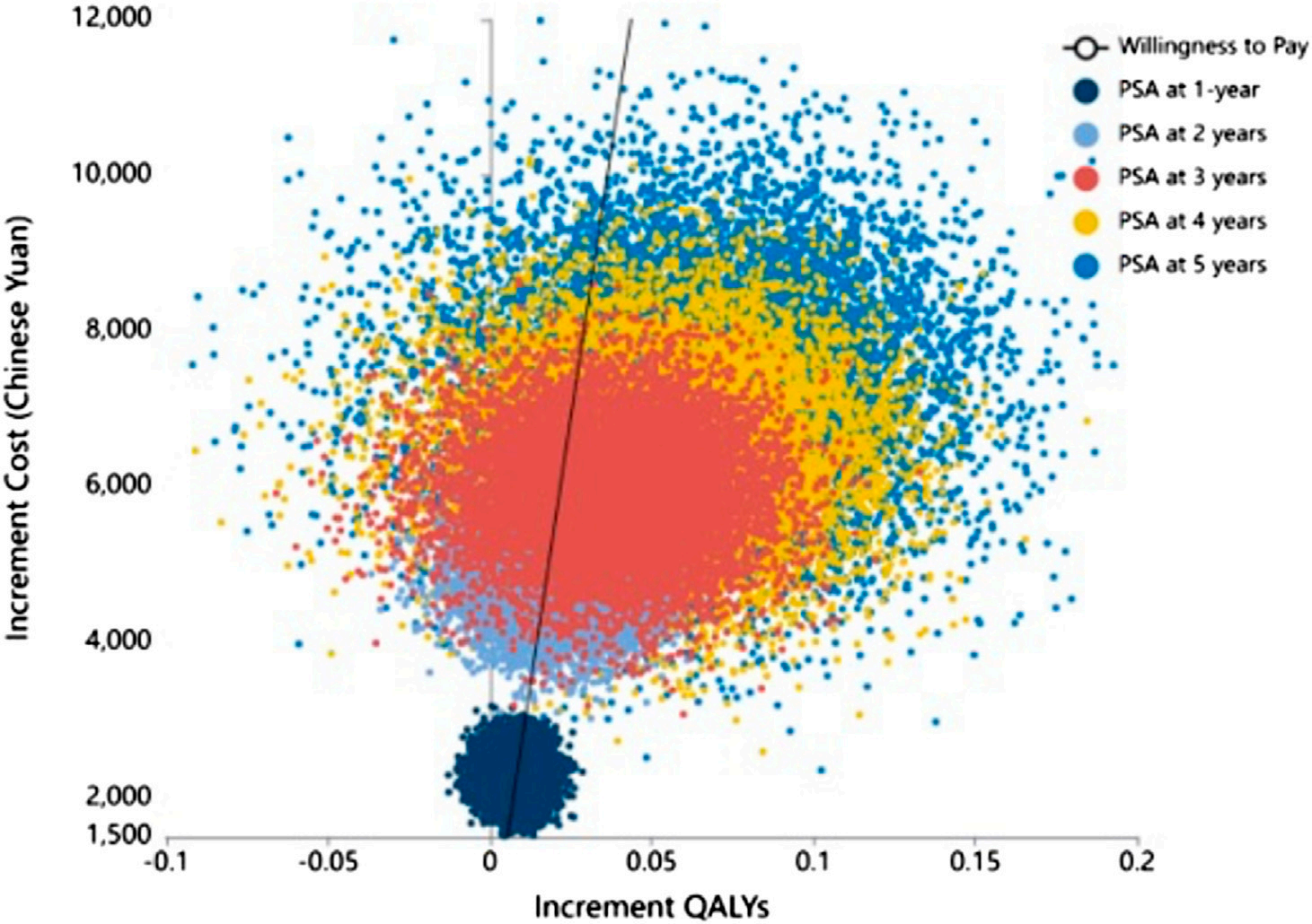
Cost-effectiveness plane showing incremental costs *versus* incremental QALY at 1, 2, 3, 4, and 5 years post-treatment. Threshold line at ¥257,094/QALY for 100,000 PSA simulations. QALY: Quality-adjusted life year.

Subsequently, we conducted a one-way sensitivity analysis of the uncertain parameters. The outcomes of this analysis were graphically depicted using a tornado diagram, revealing that the unit cost of RTX and the utility value for relapse exerted the greatest influence on the ICER (Figure 3). The cost-effectiveness plane, as shown in Figure 4, plots the incremental costs against the incremental QALYs for the durations of 1, 2, 3, 4, and 5 years post-treatment. Employing a WTP threshold of ¥257,094 per QALY, we ran a Monte Carlo simulation of 100,000 iterations. The results from this analysis indicated that the likelihood of RTX being cost-effective at 1, 2, 3, 4, and 5 years post-treatment was 46.886%, 59.172%, 73.624%, 79.182%, and 80.625%, respectively.

## 4 Discussion

RTX, administered either as monotherapy or in combination with other immunosuppressive agents, is recommended as the initial line of therapy for MN patients with a high risk of kidney injury (2021). Although both RTX and TAC are currently advocated for the treatment of MN, TAC monotherapy is regarded as less effective and is associated with a heightened relapse rate post-rapid withdrawal (Ramachandran et al., 2017; Rojas-Rivera et al., 2023). In comparison with traditional immunotherapeutic regimens, RTX shows similar efficacy but with a reduced side-effect profile. Nonetheless, its broader clinical implementation is somewhat constrained by its relatively high unit cost. Despite being a crucial clinical requirement, there remains a paucity of evidence on cost-effectiveness, especially concerning a direct comparison between RTX and other immunosuppressive medications (Barbour et al., 2018; Hamilton et al., 2018). To our knowledge, the present study is the first to assess the outcomes of RTX and TAC for MN patients in a Chinese context using a Markov model. Both cases were derived from real-world studies conducted in southern China, thereby providing a more realistic reflection of the effects of the two treatment options and the differences between them in clinical practice.

Our study indicates that RTX is a more cost-effective treatment approach compared to TAC for the management of PMN. The ICER for RTX as opposed to the TAC regimen is estimated at approximately ¥124,631.14 per QALY. When the willingness-to-pay (WTP) threshold is established at ¥257,094/QALY, RTX clearly emerges as a more economically viable option for patients afflicted with MN. The baseline analysis in our research demonstrated that the application of the RTX regimen over a 5-year duration resulted in an elevation of 0.058 QALYs and an associated cost increase of ¥7,341. The calculated ICER of RTX in contrast to TAC was ¥124,631.14/QALY, a figure that was determined to be lower than the WTP threshold of ¥257,094/QALY. Our probability sensitivity analysis, particularly the probability of the ICER being below the WTP threshold at 5 years post-treatment being 80.625%, further confirms the validity of the conclusion.

In addition to cost-effectiveness, RTX also demonstrates superior safety profiles. In the conducted safety analysis, no statistically significant difference in adverse effect rates was found between RTX (9.43%) and TAC (13.16%) treatment groups. Nevertheless, upon closely scrutinizing individual cases, it was revealed that the AEs observed in the RTX group were relatively mild in nature. These consisted of two instances of rash, one of fever, one of gastrointestinal infection, and one of pulmonary infection. Conversely, in the TAC group, beyond five cases of hyperglycemia, serious AEs were documented, including four instances of pneumonia, one of lung abscess, and one of interstitial pneumonia. Regrettably, despite rescue efforts, three patients with severe pneumonia in the TAC group succumbed to their illness, leading to a SAEs rate of 7.89% and a mortality rate of 3.95%. Hence, even though it was not explicitly underscored in the analysis and model, RTX treatment for MN presents a comparatively safer profile.

While this research is a pioneering effort at a comprehensive cost-effectiveness assessment of RTX and TAC for PMN treatment, there are certain limitations to consider. The cases analyzed for both groups originated from single-center, real-world studies in tertiary hospitals, with the potential for heterogeneity in treatment and limited follow-up periods, which may affect the findings’ accuracy and reliability. However, rigorous selection criteria were employed to mitigate potential biases. Further, the data sources for the two groups were different, resulting in a longer onset time in the RTX group compared to the TAC group. This disparity may be due to the perception that RTX, being more costly, is typically utilized when other therapies have proven ineffective. Therefore, patients who are administered RTX might initially present with more serious conditions, possibly leading to an overemphasis on TAC’s efficacy and an understated portrayal of RTX’s effectiveness. Furthermore, due to the relatively short duration of follow-up in our study, our model might underestimate the frequency of progression to ESRD over a patient’s lifetime. This underscores the importance of extended monitoring in the context of PMN. Lastly, the scope of the model was limited to treatment costs and did not account for broader societal costs like lost workdays or travel expenses, which could contribute significantly to the burden on patients, families, and society at large. Despite this potential bias, it does not alter the conclusion that RTX is more cost-effective.

## 5 Conclusion

In summary, our research provides a comprehensive and reliable pharmacoeconomic evaluation in a Chinese setting, demonstrating RTX’s superiority over TAC in terms of efficacy, safety, and cost-effectiveness in MN treatment. In the context of healthcare systems, especially those in developing nations such as China, it is essential to optimize patient care within the constraints of finite resources while considering the comprehensive health and economic benefits of diverse treatment options. Future research should focus on multi-center, large-scale, and long-term randomized controlled trials to more fully substantiate and clarify both the therapeutic and economic benefits of RTX.

## Data Availability

The original contributions presented in the study are included in the article/Supplementary Material, further inquiries can be directed to the corresponding author.
