# Supplementary material for: Comparative pharmacoeconomic analysis of rituximab and traditional tacrolimus regimens in membranous nephropathy in China
Source: Front Pharmacol. 2024 Jan 8;14:1309930. doi: 10.3389/fphar.2023.1309930 (PMC10800561; doi:10.3389/fphar.2023.1309930)
Supplement: Supplementary file 1 [file Table1.docx]

**Supplementary Table 1.** Baseline characteristics of RTX regimen group

Abbreviations: ACEI, Angiotensin-converting enzyme inhibitor; ARB, Angiotensin receptor blocker; CCB, Calcium channel blockers; Onset time: the period between the diagnosis of PMN and the initiation of treatment.

| Characteristics | RTX group |
| --- | --- |
| Patients | 53 |
| Age (year) | 31.91 ± 16.78 |
| Gender (male/female) | 41/12 |
| Onset time (month) | 41.18 ± 61.49 |
| Qualitative proteinuria test (53 episodes) | |
| - | 1 |
| ± (0.15 g/L) | 0 |
| 1 + (0.3 g/L) | 3 |
| 2 + (1 g/L) | 2 |
| 3 + (3 g/L) | 15 |
| 4 + (10 g/L) | 32 |
| Quantitative proteinuria test (g/day)（44 episodes） | |
| Mean | 7.28 ± 4.98 |
| 0-3.5 (g/day)/ | 9 |
| 3.5-4 (g/day) | 5 |
| 4-8 (g/day) | 13 |
| > 8 (g/day) | 17 |
| Serum albumin (g/L) | 39.1 ± 10.45 |
| Serum creatinine (μmol/L) | 83.16 ± 34.7 |
| Temperature (℃) | 36.48 ± 0.21 |
| Pulse rate | 96.3 ± 18.47 |
| Respiration rate | 19.98 ± 0.14 |
| Systolic blood pressure (mmHg) | 131.4 ± 17.58 |
| Diastolic blood pressure (mmHg) | 85.53 ± 14.5 |
| Co-medication |  |
| CCB | 1 |
| Diuretics | 19 |
| Lipid-lowering drugs | 29 |
| ACEI/ARB | 3 |

**Supplementary Table 2.** Definition of RTX responsiveness in PMN based on the KDIGO 2021 clinical practice guideline and the authors’ clinical experience.

| **Nephrotic syndrome (NS)** |
| --- |
| Massive proteinuria (> 3.5 g/d or urine protein-to-creatinine ratio (UPCR) > 3500 mg/g), hypoalbuminemia (< 30 g/l), hyperlipidemia, and edema. |
| **Complete remission (CR)** |
| A 24-hour urinary protein level less than 0.3g, a UPCR of less than 300mg/g, or a negative (−) or trace (+) (0.15g/L) urine protein qualitative result, all in conjunction with normal renal function. |
| **Partial remission (PR)** |
| A UPCR ranging from 300–3500mg/g, a urine protein qualitative result of 1+ (0.3g/L) or 2+(1g/L), or a decrease in proteinuria exceeding 50% compared to the pre-treatment level, yet remaining under 3.5g/24h. |
| **No Remission (NR)**  A PCR continuously exceeding 3500mg/g or a urine protein qualitative result of 3+ (3g/L) or 4+ (10g/L) after immunosuppressive treatment. |
| **Relapse**  Patients who previously attained CR and subsequently exhibited significant proteinuria post-treatment (PCR > 3500mg/g or a urine protein qualitative result > 3+). |
